# Differential Effect of Perceived Social Mobility on Sense of Gain

**DOI:** 10.3390/bs16071141

**Published:** 2026-07-07

**Authors:** Miao Lv, Yingxu Hou, Qing Yang

**Affiliations:** 1 School of Psychology, Qufu Normal University, Qufu 273165, China; houyingxu@qfnu.edu.cn (Y.H.); qyang@qfnu.edu.cn (Q.Y.); 2 Research Center for Personality Development and Education of Children and Adolescents, Qufu Normal University, Qufu 273165, China

**Keywords:** sense of gain, perceived social mobility, social trust, subjective social status

## Abstract

The subjective assessment of social mobility opportunities plays an important role in shaping individuals’ psychological experiences and social mentality. Drawing on the hierarchical structure theory of social mentality, this study examines how perceived social mobility influences sense of gain and identifies the underlying mechanisms and boundary conditions. The findings indicate the following: (1) Perceived social mobility positively predicts the sense of gain. (2) Social trust mediates the positive relationship between perceived social mobility and sense of gain, and this indirect effect is significant under high perceived social mobility but not significant under low perceived social mobility. (3) Subjective social status moderates the association between low perceived social mobility and social trust. The negative association between low perceived social mobility and social trust shows a tendency to be more pronounced among individuals with low subjective social status. These findings support an integrative model of “cognitive foundation—relational connection—emotional feedback”, providing a stratified pathway for guiding and empowering people’s sense of gain.

## 1. Introduction

In the context of economic reform, a low evaluation of social structural openness poses significant challenges to individual development. People face increasing difficulties in the labor market, giving rise to debates about “degree devaluation” and the “futility of education”. When individuals lose confidence in the link between effort and upward mobility, the “effort–reward” contract that underpins social cohesion may weaken, thereby undermining both individual well-being and social order (Davidai & Wienk, 2021). From the perspective of social belief systems, such a loss of faith reflects a shift in a core social axiom—that is, the generalized belief about the contingency between effort and social position—which shapes how individuals understand their opportunities and allocate their personal resources (Leung & Bond, 2004; Xie et al., 2022). Social axioms, together with attitudes and values, constitute the foundational elements of belief systems that influence how people respond to structural conditions and personal circumstances in their pursuit of psychological well-being.

Sense of gain is a key indicator for evaluating social governance and quality of life (Tan et al., 2020). Amid class structure transformation and economic adjustment, individuals’ evaluations of social mobility significantly affect their sense of gain (Tan & Lv, 2023). Prior studies have often treated perceived social mobility as a continuous predictor (Y. Dong & Wang, 2025; Sagioglou et al., 2019), assuming that higher perceptions correspond to greater psychological gains. However, relatively little attention has been paid to whether the psychological mechanisms linking perceived social mobility to sense of gain differ between high and low levels of perceived social mobility. This issue deserves attention because the way individuals cognitively represent the interplay between external structural conditions and their own position within the social hierarchy is central to judgments about whether valued goals are attainable (Leung & Bond, 2004; Xiao et al., 2024). Accordingly, the present study focuses not simply on whether perceived social mobility is related to sense of gain, but on whether this relationship operates differently under conditions of high versus low perceived mobility.

Existing literature shows that positive evaluations of mobility opportunities enhance trust, expand support networks, and improve overall well-being (Day & Fiske, 2017; Feng & Zhong, 2021; Ren et al., 2022). However, many studies treat low perceived social mobility as the direct opposite of high perceived social mobility, neglecting possible differences in the underlying psychological processes. When individuals perceive social structures as closed, they may adopt defensive strategies (Hobfoll et al., 2018), which could hinder the pathway linking mobility perception to sense of gain via social trust.

To address this gap, this study draws on the hierarchical structure theory of social mentality, positioning perceived social mobility at the “cognitive foundation” level, sense of gain at the “emotional feedback” level, and social trust as a dual pathway at the “relational connection” level (M. Chen, 2024). Within this framework, perceived resource availability may shape individuals’ perceptions of social situations and future development, thereby affecting social trust (Brandt et al., 2015; Hobfoll et al., 2018), which may in turn be associated with their sense of gain. Through a cross-sectional survey of 372 adults (aged 18–45) from provinces such as Guangdong and Shandong, and an experimental study with 188 adults (aged 18–45) from provinces such as Shandong and Jiangsu, we examined whether the effects of high and low social mobility perceptions differ across the mediating pathway. We also introduced subjective social status as a moderating variable to examine whether available resources buffer the potential negative consequences of low perceived social mobility. For individuals with low perceived social mobility, lower subjective social status may intensify negative psychological responses due to social cognitive patterns and insufficient resources (Kraus et al., 2009; Stephens et al., 2014), weakening social trust and, in turn, sense of gain. Taken together, the present study seeks to clarify whether the relationship between perceived social mobility and sense of gain differs between high and low perception conditions, and whether this difference is conditioned by subjective social status.

Compared with prior studies, this research addresses several gaps in the existing literature. First, unlike prior research that has primarily treated perceived social mobility as a linear predictor, it examines whether the indirect effect of perceived social mobility on sense of gain via social trust differs between high and low levels of perceived mobility. Second, it introduces social trust as a relational mechanism, complementing the predominant focus on individual-level processes in prior work. Third, by incorporating subjective social status, it identifies for whom perceived social mobility matters most. Together, these findings offer a more nuanced understanding of how perceived social mobility shapes individuals’ sense of gain. Theoretically, this study contributes to the “cognitive foundation—relational connection—emotional feedback” framework by identifying social trust as a mediating mechanism and by showing that this indirect pathway operates only under high perceived social mobility. Practically, it provides empirical support for differentiated intervention strategies, suggesting that policies aimed at enhancing people’s sense of gain should move beyond one-size-fits-all approaches and instead be tailored to the resource positions of specific subgroups.

### 1.1. The Impact of Perceived Social Mobility on Sense of Gain

Perceived social mobility is a key construct in social mentality, reflecting individuals’ subjective assessment of social class openness and mobility opportunities (Day & Fiske, 2017; Kraus & Keltner, 2013). Social axioms are fundamental premises about how the world operates, functioning alongside attitudes and values to orient behavior across situations (Bond et al., 2004). Within the broader framework of social belief systems, perceived social mobility can be understood as a social axiom: whether society is seen as an open structure in which effort yields advancement, or a closed one in which positions remain fixed regardless of individual striving. This perception is not merely a reflection of objective conditions; it is shaped by cognitive frameworks that influence belief systems and behavior. Empirical studies show that high perceptions of social mobility foster future-oriented thinking, enhancing life meaning and overall well-being (Gugushvili, 2022; Schneider, 2012; Lin et al., 2022). Furthermore, individuals with high mobility perceptions tend to view social systems as open, increasing their support for institutional legitimacy and contributing to social stability (Day & Fiske, 2017; Sagioglou et al., 2019). On a macro level, these perceptions drive the public to pursue a higher quality of life and engage in socioeconomic activities (Tan & Lv, 2023). Given the impact of dynamic social environments on individuals’ mentality (Liu et al., 2022), perceived social mobility, as a core social axiom, plays a critical role in shaping variations in sense of gain.

Sense of gain belongs to a higher level of social mentality, referring to individuals’ cognitive evaluations of the content, pathways, and conditions of need satisfaction, accompanied by relatively stable positive psychological experiences (H. Dong et al., 2019). The concept of “sense of gain” was originally proposed in the Chinese context as an indicator of how citizens subjectively evaluate the benefits of social and economic development. Unlike traditional conceptions of well-being, sense of gain emphasizes individuals’ integrated evaluation of objective gains and subjective experience. Its enhancement depends not only on support from the social environment but also on individuals’ active participation in the process of obtaining those gains (Tan et al., 2020). At the individual level, sense of gain is significantly positive correlated with well-being, life satisfaction, and sense of security (Feng & Zhong, 2021), constituting a key variable driving individuals to pursue high-quality development (Tan et al., 2020). At the macro level, enhancing sense of gain can reduce negative social emotions, optimize institutional trust and risk expectations, and thereby reinforce social stability (Ji & Hu, 2022). Therefore, enhancing individuals’ sense of gain is not only directly related to their well-being but also serves as a strategic pivot for cultivating positive social mentality and promoting common prosperity (Xiang & Ma, 2022).

The positive impact of perceived social mobility on sense of gain is evident. High perceived social mobility stabilizes social mentality, alleviating resentment and hostility arising from status disadvantages, reinforcing beliefs in a just world (Day & Fiske, 2017; Sagioglou et al., 2019), and enhancing sense of gain through increased fairness perceptions (Y. Yang & Ni, 2025). When individuals believe they can achieve status change through effort, their sense of control increases, leading to more positive social attitudes (L. Li et al., 2018). Multiple studies confirm that improvements in perceived social mobility significantly enhance sense of gain (Tan & Lv, 2023; Y. Wang et al., 2020). Therefore, this paper proposes

**Hypothesis 1** **(H1).** *Perceived social mobility positively predicts sense of gain.*

### 1.2. Mechanisms of the Impact of Perceived Social Mobility on Sense of Gain

Based on the hierarchical structure theory of social mentality (M. Chen, 2024), this study constructs a concise “cognitive foundation—relational connection—emotional feedback” framework to examine how perceived social mobility may relate to sense of gain through social trust. Within this framework, perceived social mobility is defined as a subjective assessment of opportunities for upward social mobility and constitutes the foundational level of social mentality. Sense of gain refers to individuals’ cognitive, emotional, and behavioral evaluations of whether their needs are being satisfied during social reform and is positioned at a higher level of social mentality (Tan et al., 2020). Following this hierarchical logic, social trust serves as a key mediator linking foundational cognition and advanced emotions, thereby constituting a secondary level of social mentality.

Social trust is defined as individuals’ general expectation of the trustworthiness of others and serves as an indicator of social harmony (Putnam, 1995). This trust is rooted in cognitive tendencies shaped by institutional guarantees and risk assessments rather than by specific interpersonal interactions (Schilke et al., 2021). Institutional trust underpins social trust; effective institutions reduce transaction uncertainty by creating a shared cognitive framework (Luhmann, 1979; Delhey et al., 2011). Although institutional trust provides a systemic backdrop, perceived fairness reflects judgments of justice, and meritocratic beliefs offer a motivational rationale, the present study focuses on social trust as the relational mechanism because it more directly captures the interpersonal resource that links cognitive foundations to emotional feedback. Empirical studies show that higher social trust alleviates anxiety and uncertainty, improves mental health, enhances subjective well-being, and increases sense of gain (Feng & Zhong, 2021; Lu et al., 2020; Y. Wang et al., 2020).

To explain why this process may differ between high and low perceived social mobility, we integrate resource conservation theory (Hobfoll, 1989). Resource conservation theory suggests that individuals are especially sensitive to potential resource loss and tend to adopt strategies that protect existing resources when they perceive threat or scarcity (Hobfoll et al., 2018). Under conditions of high perceived social mobility, individuals are more likely to believe that effort can lead to class transition and sustained access to developmental resources. Individuals with greater social resources are also better able to absorb the costs of trusting others, which increases social trust (Hamamura, 2012; Navarro-Carrillo et al., 2018). Those with high perceived social mobility often hold stronger fairness beliefs, which further promotes trust (Day & Fiske, 2017; Ren et al., 2022). Elevated social trust, in turn, facilitates a stronger sense of gain (Feng & Zhong, 2021). Through this pathway, the cognitive evaluation of open mobility is translated into a positive emotional experience. By contrast, low perceived social mobility fosters beliefs about blocked opportunities and entrenched inequality. In such circumstances, trusting others is perceived as riskier and less likely to be reciprocated with valuable resources, leading individuals to withhold trust in order to protect their existing psychological assets (Hobfoll et al., 2018). This defensive orientation reduces willingness to extend trust and thereby weakens the association between perceived social mobility and sense of gain.

Based on this reasoning, we propose

**Hypothesis 2** **(H2).** *Social trust mediates the positive relationship between perceived social mobility and sense of gain, and this indirect effect is significant under high perceived social mobility but not significant under low perceived social mobility.*

### 1.3. The Moderating Role of Subjective Social Status

Research typically views low subjective social status as a key risk factor for mental health and social welfare (Jackson et al., 2015; O’Leary et al., 2021). Yet static indicators of class position may fail to capture the extent to which individuals experience closure in relation to upward mobility. Low perceived social mobility may better predict a lack of sense of gain than static class position, but empirical evidence remains limited. This study therefore examines whether low perceived social mobility inhibits sense of gain through social trust.

Previous studies confirm that subjective social status influences the mechanisms underlying perceived social mobility (Rheinschmidt & Mendoza-Denton, 2014; Xiao et al., 2024). Subjective social status is the self-assessment of one’s social position through social comparison and directly shapes psychological and behavioral responses (Kraus et al., 2012). This self-classification affects the experiences and behaviors of individuals across class groups, with class differences primarily rooted in resource availability (Stephens et al., 2014). Those with high subjective social status enjoy abundant resources, lower psychological stress, and significantly better mental health, well-being, and life satisfaction (Demakakos et al., 2008; Sakurai et al., 2010). Conversely, individuals with low subjective social status perceive resource scarcity and competitive disadvantages, leading to stress and higher rates of negative emotions such as anxiety and depression (Dohrenwend & Dohrenwend, 1981; Madigan & Daly, 2023), as well as lower life satisfaction (Main & Bradshaw, 2012).

Subjective social status may moderate the relationship between perceived social mobility and social trust. This moderating role can be understood as a manifestation of the cognitive interplay between subjectively perceived environmental constraints and personal constraints. Research shows that when perceived social mobility is low, subjective social status is positively correlated with subjective well-being; this correlation weakens when perceived social mobility is high (Huang et al., 2017). W. Li et al. (2020) noted that high perceived social mobility generally boosts life satisfaction across social statuses. It fosters the belief that effort leads to change and enhances confidence in the future. This hope can mitigate the negative effects of resource scarcity or status anxiety, thereby stabilizing social trust. We therefore hypothesize that subjective social status does not affect the impact of high perceived social mobility on social trust, but the negative association between low perceived social mobility and social trust may be more pronounced among individuals with lower subjective social status.

Social cognitive theory explains class differences among individuals with low perceived social mobility through attribution styles. Those with high subjective social status tend to adopt an individualistic orientation, attributing outcomes to internal, controllable factors, which enhances their sense of control and fairness (Kraus et al., 2009; S. Yang et al., 2016). Even when facing reduced mobility opportunities, they may externalize the issue as a broader social phenomenon, potentially buffering negative impact on social trust through their stronger sense of control. Conversely, individuals with low subjective social status adopt a situational orientation, are more sensitive to external threats, and experience lower perceived control and self-efficacy (Barbareschi et al., 2008; Kraus et al., 2012). When confronted with blocked social mobility, they are more likely to attribute the causes to stable, uncontrollable external factors (Kraus et al., 2009). This external attribution, combined with the belief that “effort is futile”, can reduce control and self-efficacy, which may further weaken social trust (Kraus & Keltner, 2013).

Moreover, differences in resource possession offer another perspective on how low perceived social mobility affects social trust differently across levels of subjective social statuses. Individuals with high subjective social status possess more material and social capital (Stephens et al., 2014) and maintain higher social trust even when they perceive limited mobility opportunities, partly due to greater risk tolerance. In addition, their internal control tendencies enable them to mobilize resources effectively, reducing feelings of powerlessness caused by low perceived social mobility. In contrast, low perceived social mobility combined with low subjective social status may be associated with an even greater reduction in social trust. Individuals in lower-status positions, often exposed to prolonged stress, experience continuous depletion of psychosocial resources (Gallo & Matthews, 2003). After upward social comparisons, they are more likely to experience negative emotions (Cramer et al., 2016), which could further intensify the adverse effects of low perceived social mobility on social trust. Taken together, when low perceived social mobility signals a closed environment, low subjective social status signals limited personal resources to cope with that closure; it is the joint evaluation of these two constraints that shapes the decision to withhold social trust.

Based on this, we propose

**Hypothesis 3** **(H3).** *Subjective social status moderates the link between low perceived social mobility and social trust. Specifically, individuals with a low subjective social status demonstrate a stronger negative impact of low perceived social mobility on social trust.*

## 2. Study 1: Questionnaire Survey

Study 1 investigates the association between perceived social mobility on the sense of gain, as well as the indirect effect of social trust in this relationship.

### 2.1. Participants

A total of 379 valid participants were recruited through the Credamo platform, a Chinese online data collection platform widely used in psychology and behavioural research, with many published studies relying on its services (H. Chen et al., 2025; Ye & Li, 2025). Recruitment criteria specified mainland Chinese residents aged 18–45. This age group represents early to mid-adulthood, a life stage in which perceptions of social mobility are especially relevant. Participants who failed the attention check (item 15: “Please select 4 for neutral”) were excluded. No missing data were present for the key variables, as the online platform enforced response requirements. After screening, 372 responses from adults (aged 18 to 45) were obtained, including 174 males and 198 females. Detailed demographic characteristics are provided in Appendix A Table A1. Upon completion, participants received a reward of 5 RMB.

### 2.2. Research Materials and Tools

#### 2.2.1. Perceived Social Mobility

The Social Mobility Beliefs Scale developed by Sagioglou et al. (2019) was used in this study. This unidimensional scale consists of 7 items and is scored on a 7-point Likert scale, ranging from 1 (strongly disagree) to 7 (strongly agree). An example item is as follows: “The social environment we are born into determines our entire life.” Items 1, 2, and 5 are reverse-scored, with higher scores indicating stronger perceived social mobility. In this study, the Cronbach’s alpha coefficient for this scale was 0.78.

#### 2.2.2. Sense of Gain

The Sense of Gain Scale developed by H. Dong et al. (2019) was used in this study. This multidimensional scale comprises five subscales—experience of gain, environment of gain, content of gain, pathways of gain, and sharing of gain. The scale consists of 15 items and is scored on a 7-point Likert scale, ranging from 1 (strongly disagree) to 7 (strongly agree), with higher scores indicating a stronger sense of gain. An example item is: “The inclusiveness and friendliness of society make my life more fulfilling.” In this study, the Cronbach’s alpha coefficient for this scale was 0.92.

#### 2.2.3. Social Trust

Social trust was assessed using four items developed by Tan (2016): “People are, in most cases: A. Helpful; B. Self-interested and indifferent to others”, “Most people in society: A. Can be trusted; B. Should be approached with caution”, “Most people in society will: A. Treat others as fairly as possible; B. Take advantage of others whenever possible”, and “Most people in society trust strangers”. The first three items were reverse-scored, employing a 7-point Likert scale (from “1 = Strongly Agree A” to “4 = Neutral” to “7 = Strongly Agree B”); the fourth item also utilized a 7-point Likert scale (from “1 = Strongly Disagree” to “7 = Strongly Agree”). The average score of these four items was calculated as the score for social trust, with higher scores indicating greater social trust. The Cronbach’s α coefficient of the scale in this study was 0.85.

#### 2.2.4. Demographic Variables

Subjective social status was measured using the item “Which level do you think you currently occupy?” from the MacArthur Scale of Subjective Social Status (Adler et al., 2000). Participants viewed a 10-level social ladder representing societal class positions, where a higher rung indicates greater wealth, education, and living conditions. For example, level 1 represents the very bottom of the social ladder, where people experience the worst living conditions—the lowest level of education, the least decent jobs, and the lowest income. Level 10 represents the very top of the social ladder, where people enjoy the most affluent living conditions—high levels of education, the most decent jobs, and the highest income. Participants were asked to select their position on this ladder. The full list of items for all scales used in Studies 1 and 2 is provided in the Supplementary Materials.

Additional control variables included demographic information such as gender, age, and monthly family income.

### 2.3. Results

#### 2.3.1. Common Method Bias Test

Common method bias in the data was examined using the “Harman’s Single Factor Test”, and the “Factor Model Comparison Method” (Tang & Wen, 2020). First, Harman’s single factor test indicated that there were 5 factors with eigenvalues greater than 1, and the first factor explained 35.844% of the variance, which is below the critical threshold of 40.00%. Second, a comparison was made between a single factor model (method factor) and a three-factor model (perceived social mobility, sense of gain, and social trust). The results showed that the three-factor model significantly outperformed the single factor model (Δχ^2^/Δdf = 49.692, *p* < 0.001, ΔCFI = 0.248, ΔTLI = 0.228, ΔRMSEA = −0.038). These results suggest that there is no significant common method bias in this study.

#### 2.3.2. Descriptive Statistics and Correlation Analysis

Table 1 presents the means and standard deviations for the main variables in Study 1. The sense of gain was significantly positively correlated with perceived social mobility and social trust (*p* < 0.001). Perceived social mobility was significantly positively correlated with social trust (*p* < 0.001).

#### 2.3.3. Mediation Analysis

**Figure 1:**
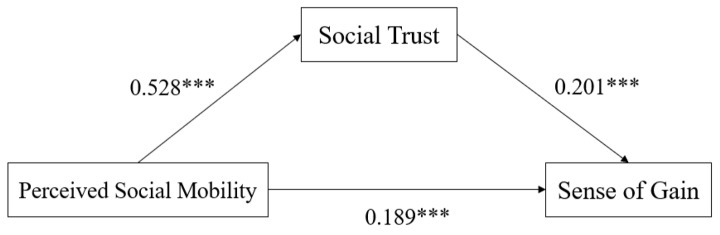
The mediation model of social trust in study 1 (*n* = 372). Notes: *** *p* < 0.001.

Model 4 from the SPSS version 25.0 macro PROCESS developed by Hayes (2013) was employed with 5000 bootstrap samples to analyse the mediating effect of social trust on the relationship between perceived social mobility and sense of gain. The results (see Table 2 and Figure 1) indicated that perceived social mobility was significantly positively associated with social trust ($a_{1}$ = 0.528, *SE* = 0.059, *p* < 0.001). When the mediating variable was introduced, the association between social trust and sense of gain remained significant ($b_{1}$ = 0.201, *SE* = 0.033, *p* < 0.001), while the direct association between perceived social mobility and sense of gain also retained significance (*c’* = 0.189, *SE* = 0.041, *p* < 0.001). The indirect effect was significant ($a_{1}b_{1}$ = 0.106, *Boot SE* = 0.027, 95% CI = [0.055, 0.161]), indicating that the mediation model through social trust was supported. Monte Carlo simulation results showed that the indirect effect was significant, with estimated statistical power of 1.00, indicating a robust mediation effect.

In addition to the standard mediation analysis, we further examined whether the indirect effect of perceived social mobility on sense of gain via social trust differed between low and high levels of perceived mobility. Perceived social mobility was categorized into three groups using the upper 27%, middle 46%, and lower 27% of the sample distribution. The same mediation framework was then applied to these grouped data. Referring to the mediation analysis method for multiple categorical independent variables (Fang et al., 2017; Zhao et al., 2025), the analysis was conducted using Model 4 of PROCESS version 3.3 with 5000 bootstrap samples. Condition was dummy coded into two independent variables, with the control group as the reference group: low perceived social mobility (low condition = 1, high condition = 0, control condition = 0) and high perceived social mobility (low condition = 0, high condition = 1, control condition = 0). Social trust was added as the mediator and sense of gain was added as the dependent variable, with gender, age, monthly family income, and subjective social status included as covariates (see Table 3).

**Figure 2:**
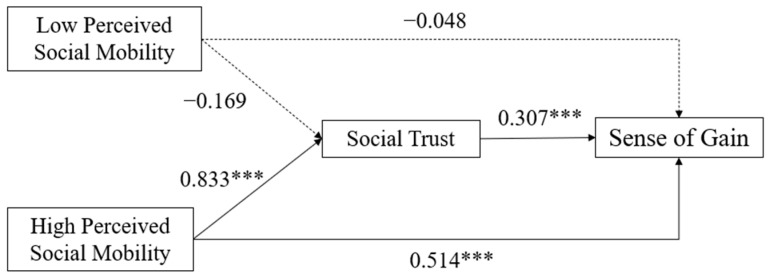
The mediation pathways of social trust (*n* = 372). Notes: All path coefficients are standardized; Solid arrows indicate significant paths, and dashed arrows indicate non-significant paths; *** *p* < 0.001.

The results of the overall mediation analysis indicate that the test for the total effect yields *F*_(2, 365)_ = 28.944, *p* < 0.001, suggesting that the two relative total effects are not all zero. The test for the overall direct effect yields *F*_(2, 364)_ = 11.520, *p* < 0.001, indicating that the two relative direct effects are significant. Figure 2 illustrates the standardized coefficients of the model. The direct path from high perceived social mobility to sense of gain was significant (β = 0.770, *p* < 0.001), whereas the direct path from low perceived social mobility to sense of gain was not (β = −0.100, *p* = 0.361). Importantly, there was a significant indirect path from high perceived social mobility to sense of gain through social trust (β = 0.256, 95% CI = [0.126, 0.407]). A non-significant indirect path was found from low perceived social mobility to sense of gain through social trust (β = −0.052, 95% CI = [−0.148, 0.025]). This pattern supports the hypothesized differential mechanism, whereby high perceived social mobility operates through social trust to enhance sense of gain, whereas this pathway is absent under low perceived social mobility.

## 3. Pre-Experiment: Manipulation Specificity of Perceived Social Mobility

Although the perceived social mobility manipulation used in Study 2 was adapted from an established paradigm (Day & Fiske, 2017), it remained possible that the manipulation might inadvertently affect related constructs such as belief in a just world, relative deprivation, risk perception, or social expectation. To address this concern, we conducted a pre-experiment prior to Study 2 to examine whether perceived social mobility was significantly associated with these variables. If perceived social mobility was not significantly related to these constructs, this would provide preliminary evidence for the discriminant validity and specificity of the manipulation.

### 3.1. Participants

A separate sample of 44 adults (aged 18–45, 13 males, 31 females) recruited via Credamo participated in the pre-experiment. The recruitment and exclusion criteria were the same as those in Study 1. No missing data were present for the key variables, as the online platform enforced response requirements.

### 3.2. Research Materials and Tools

#### 3.2.1. Perceived Social Mobility

**Figure A1:**
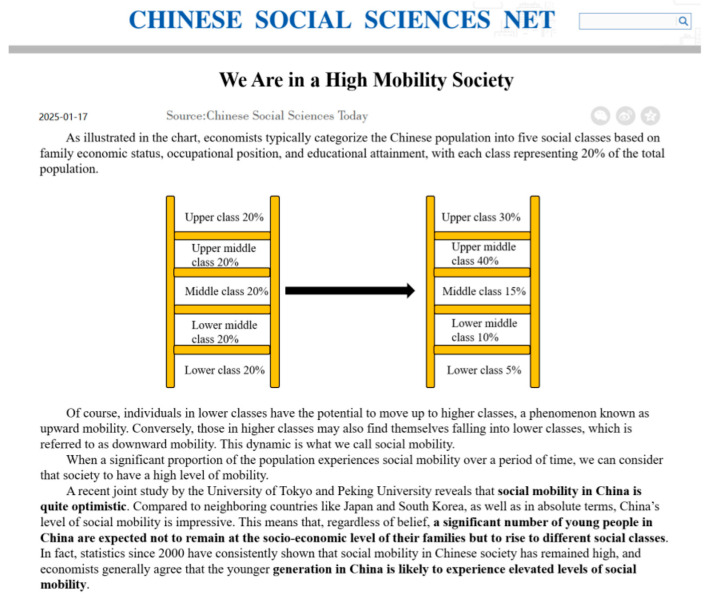
Materials for manipulating high perceived social mobility. The material was fabricated by the authors for experimental purposes and does not represent the views of any real institution.

**Figure A2:**
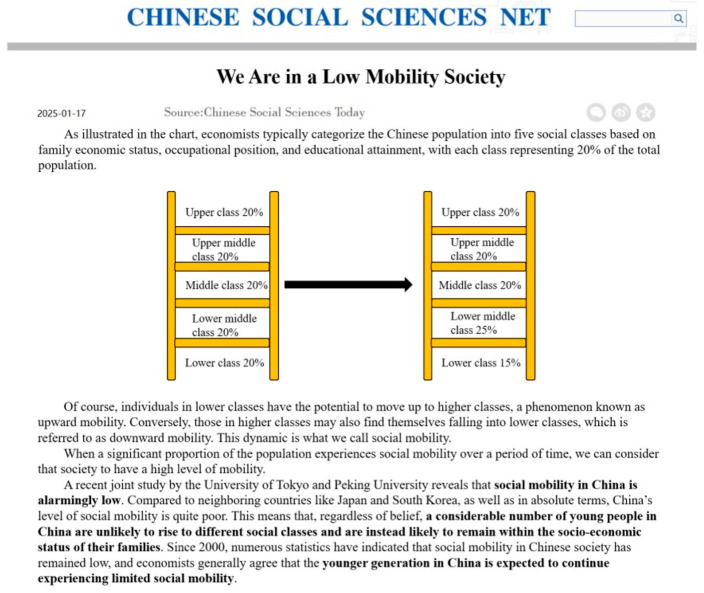
Materials for manipulating low perceived social mobility. The material was fabricated by the authors for experimental purposes and does not represent the views of any real institution.

The manipulation of perceived social mobility was performed using the method developed by Day and Fiske (2017). Participants were randomly assigned to a high perceived social mobility group (*n* = 22), and a low perceived social mobility group (*n* = 22). The high mobility group read an article titled “We Are in a Highly Mobile Society” from the Chinese Academy of Social Sciences Journal (see Appendix A Figure A1), highlighting positive social mobility in China. The low mobility group read “We Are in a Low Mobility Society” from the same journal (see Appendix A Figure A2), depicting negative social mobility. Participants then answered two questions to assess the effectiveness of the manipulation: “The social environment we are born into determines our entire life” and “In today’s society, it is difficult for a person’s social status to improve throughout their life”. Both statements were rated on a 7-point scale (from “1 = strongly disagree” to “7 = strongly agree”), with both items scored in reverse; a higher score indicates a higher level of perceived social mobility.

#### 3.2.2. Belief in a Just World

Belief in a just world was assessed using the 13-item Belief in a Just World Scale developed by Dalbert (1999) and revised by Su et al. (2012). The scale includes two dimensions: general belief in a just world and personal belief in a just world. A representative item is: “I think the world is basically just.” All items were rated on a 7-point Likert scale, ranging from 1 (strongly disagree) to 7 (strongly agree), with higher scores indicating a stronger belief in a just world. In this study, the Cronbach’s alpha coefficient was 0.95.

#### 3.2.3. Relative Deprivation

Relative deprivation was measured using the 4-item Relative Deprivation Scale developed by Ma (2012). A representative item is: “Compared with my efforts and contributions, my life should be better than it is now.” The scale uses a 7-point Likert scale, ranging from 1 (severely disagree) to 7 (significantly agree), with higher scores indicating a higher level of relative deprivation. In this study, the Cronbach’s alpha coefficient was 0.80.

#### 3.2.4. Risk Perception

Risk perception was assessed using a 7-item scale that asked participants to rate the severity of various social problems in China. Participants responded to the question: “In your opinion, how serious are the following problems in China?” on a 7-point scale ranging from 1 (not serious at all) to 7 (very serious). The items covered environmental problems, income inequality, and related issues. In this study, the Cronbach’s alpha coefficient was 0.93.

#### 3.2.5. Social Expectation

Social expectation was measured using a 4-item scale asking participants to predict changes over the next five years in income level, debt situation, job prospects, and personal development. Participants responded on a 7-point scale ranging from 1 (greatly decrease) to 7 (greatly increase). The debt item was reverse-scored. In this study, the Cronbach’s alpha coefficient was 0.83.

### 3.3. Research Procedure

The experiment began with the perceived social mobility manipulation. Next, a manipulation check was conducted to verify its effectiveness. Participants then completed the belief in a just world, relative deprivation, risk perception, and social expectation scales.

### 3.4. Results

#### 3.4.1. Manipulation Check

An independent samples t-test was conducted to evaluate the manipulation check results for the high and low perceived social mobility groups. The results indicated that the high perceived social mobility group (*M* = 6.023, *SD* = 0.626) exhibited significantly higher perceived social mobility than the low perceived social mobility group (*M* = 2.364, *SD* = 0.727, *t* = 17.889, *p* < 0.001). This finding confirms that the manipulation of perceived social mobility was successful.

To further examine whether the perceived social mobility manipulation exerted unintended effects on related psychological constructs, independent samples t-tests were conducted to compare the high and low perceived social mobility conditions on belief in a just world, relative deprivation, risk perception, and social expectation. The results showed that the two conditions did not differ significantly in belief in a just world, *t* = −1.059, *p* = 0.296; relative deprivation, *t* = 0.573, *p* = 0.570; risk perception, *t* = 1.289, *p* = 0.204; or social expectation, *t* = −0.377, *p* = 0.708. These null findings suggest that the manipulation primarily altered perceived social mobility, rather than these alternative psychological states.

#### 3.4.2. Descriptive Statistics and Correlation Analysis

Pearson correlation analyses revealed that perceived social mobility was not significantly correlated with any of the four candidate confounding variables: belief in a just world (*r* = 0.161, *p* = 0.296), relative deprivation (*r* = −0.088, *p* = 0.570), risk perception (*r* = −0.195, *p* = 0.204), or social expectation (*r* = 0.147, *p* = 0.340). All correlations were small in magnitude and statistically non-significant, suggesting that perceived social mobility is empirically distinct from these constructs (see Table 4).

Overall, these findings provide preliminary evidence that the perceived social mobility manipulation used in Study 2 primarily captured perceived social mobility rather than these alternative psychological states. This strengthens the interpretation that the observed effects on social trust and sense of gain are specific to perceived social mobility.

## 4. Study 2: The Differential Path Mechanism of Perceived Social Mobility on Sense of Gain

Because Study 1 relied on self-reported survey data, it primarily provides correlational evidence for the proposed model. To strengthen causal inference, Study 2 adopts an experimental design in which perceived social mobility is manipulated to test whether changes in perceived mobility influence sense of gain through social trust. In addition, a pre-experiment was conducted to examine the specificity of the manipulation and rule out alternative explanations. Moreover, Study 2 further examines whether the effects of high and low perceived social mobility differ by distinguishing these conditions and testing their indirect effects separately.

### 4.1. Participants

A one-factor between-subjects design was utilized, and the necessary sample size for this study was determined to be 159 using G*Power 3.1.9.7 software (α = 0.05, power (1 − β err prob) = 0.8). The survey was administered online via Credamo. Initially, 196 responses were collected; after excluding participants who failed the attention check items, 188 valid responses remained. The recruitment and exclusion criteria were the same as those in Study 1. No missing data were present for the key variables, as the online platform enforced response requirements. Among the participants, 72 were male, and 116 were female, with ages ranging from 18 to 45 years. Detailed demographic characteristics are provided in Appendix A Table A2. Upon completion, participants received a reward of 5 RMB. Study 2 participants were recruited separately, and none had participated in Study 1.

### 4.2. Research Materials and Tools

#### 4.2.1. Perceived Social Mobility

**Figure A3:**
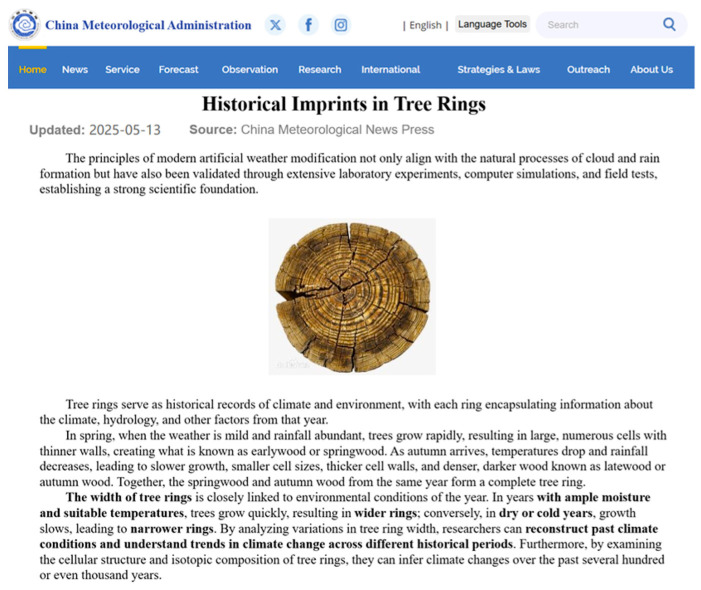
Manipulation materials for the control group. The material was fabricated by the authors for experimental purposes and does not represent the views of any real institution.

The manipulation of perceived social mobility was performed using the method developed by Day and Fiske (2017), which has been widely adopted in subsequent research on this topic (Y. Dong & Wang, 2025; Sagioglou et al., 2019). Participants were randomly assigned to a high perceived social mobility group (*n* = 62), a low perceived social mobility group (*n* = 64), and a control group (*n* = 62). The high mobility group read an article titled “We Are in a Highly Mobile Society” from the Chinese Academy of Social Sciences Journal (see Appendix A Figure A1), highlighting positive social mobility in China. The low mobility group read “We Are in a Low Mobility Society” from the same journal (see Appendix A Figure A2), depicting negative social mobility. The control group read “Historical Imprints in Tree Rings” from the China Meteorological Popular Science Network (see Appendix A Figure A3), unrelated to social mobility. Participants then answered two questions to assess the effectiveness of the manipulation: “The social environment we are born into determines our entire life” and “In today’s society, it is difficult for a person’s social status to improve throughout their life”. Both statements were rated on a 7-point scale (from “1 = strongly disagree” to “7 = strongly agree”), with both items scored in reverse; a higher score indicates a higher level of perceived social mobility.

#### 4.2.2. Sense of Gain

The Sense of Gain Scale developed by Shao (2019) was used in this study, which is a five-dimensional scale comprising political construction, economic construction, social construction, cultural construction, and ecological construction. This scale consists of 15 items and is scored on a 5-point Likert scale, ranging from 1 (severely deteriorated) to 5 (significantly improved), with higher scores indicating a stronger sense of gain. An example item is: “Anti-corruption and integrity promotion”, which participants rated following the stem: “In the past five years, what changes have you observed regarding the achievements of our country’s reform and opening-up, as well as local development and construction?” In this study, the Cronbach’s alpha coefficient for this scale was 0.92.

#### 4.2.3. Social Trust

Same as in Study 1. In this study, the Cronbach’s alpha coefficient was 0.86.

#### 4.2.4. Demographic Variables

Same as in Study 1.

### 4.3. Research Procedure

The experimental process began with the manipulation of perceived social mobility. Subsequently, a manipulation check was conducted to verify its effectiveness. Next, participants were administered the social trust scale and the sense of gain scale in sequence. Finally, participants completed the subjective social status scale and provided the necessary demographic information. After data collection, participants were given the following statement: “The article on social class mobility you read is completely fictional and was fabricated by the research team for experimental purposes. It has no connection to any official statistics, nor does it represent real data, the position of the Chinese Academy of Social Sciences, or any other institution. If you have any questions, please feel free to contact our team. Thank you again for your understanding and cooperation.” We attached our team’s contact information below the debriefing statement.

### 4.4. Results

#### 4.4.1. Manipulation Check

**Figure 3:**
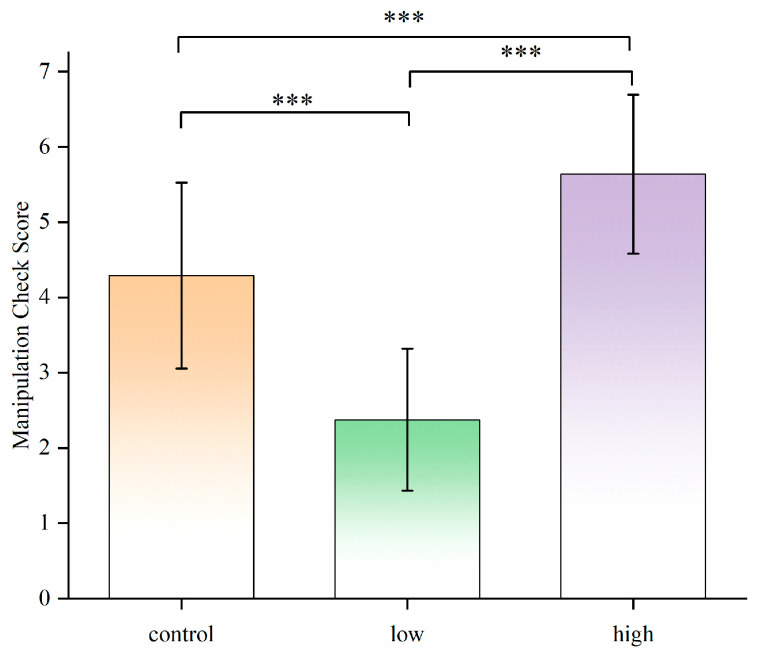
Manipulation check of perceived social mobility (*n* = 188). Notes: *** *p* < 0.001. Error bars represent standard deviation (SD). Orange bars represent the control group, green bars represent the low perceived social mobility group, and purple bars represent the high perceived social mobility group.

A one-way ANOVA was performed to assess the manipulation check results for the high and low social mobility perception groups, as well as the control group. The analysis revealed significant differences in the manipulation check scores among the three groups, *F*_(2, 185)_ = 144.65, *p* < 0.001, η^2^ = 0.61. Subsequent post hoc analysis indicated that the high perceived social mobility group (*M* = 5.64, *SD* = 1.056) scored significantly higher on social mobility perception than the control group (*M* = 4.29, *SD* = 1.233, *md* = 1.35, *p* < 0.001, 95% CI = [0.877, 1.817], Cohen’s *d* = 1.2) and the low perceived social mobility group (*M* = 2.38, *SD* = 0.943, *md* = 3.26, *p* < 0.001, 95% CI = [2.796, 3.728], Cohen’s *d* = 3.3). Additionally, the low perceived social mobility group exhibited significantly lower social mobility perception compared to the control group (*md* = −1.92, *p* < 0.001, 95% CI = [−2.381, −1.449], Cohen’s *d* = 1.7) (see Figure 3).

#### 4.4.2. Mediation Analysis

Table 5 presents the means and standard deviations for the main variables in Study 2. The sense of gain was significantly positively correlated with perceived social mobility (*p* < 0.05) and social trust (*p* < 0.001). Perceived social mobility was significantly positively correlated with social trust (*p* < 0.001).

In prior research on social mobility perception, participants have often been randomly assigned to high and low social mobility perception groups (Day & Fiske, 2017; Y. Dong & Wang, 2025; Sagioglou et al., 2019). Consequently, we initially selected data from these two groups, coding the high social mobility perception group as 1 and the low social mobility perception group as 0.

**Figure 4:**
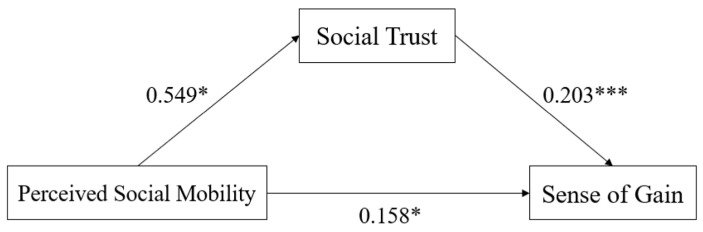
The mediation model of social trust in study 2 (*n* = 126). Notes: * *p* < 0.05, *** *p* < 0.001.

Model 4 from the SPSS version 25.0 macro PROCESS was employed with 5000 bootstrap samples to analyse the mediating effect of social trust on the relationship between perceived social mobility and sense of gain. The results (see Table 6 and Figure 4) indicated that perceived social mobility had a significant positive predictive effect on social trust ($a_{1}$ = 0.549, *SE* = 0.217, *p* < 0.05). When the mediating variables were introduced, the predictive effect of social trust on sense of gain remained significant ($b_{1}$ = 0.203, *SE* = 0.031, *p* < 0.001), while the direct predictive effect of perceived social mobility on sense of gain also retained significance (*c′* = 0.158, *SE* = 0.076, *p* < 0.05). The indirect effect of social trust was significant ($a_{1}b_{1}$ = 0.111, *Boot SE* = 0.053, 95% CI = [0.023, 0.230]).

The analysis was conducted using Model 4 of PROCESS version 3.3 with 5000 bootstrap samples. Condition was dummy coded into two independent variables, with the control group as the reference group: low perceived social mobility (low condition = 1, high condition = 0, control condition = 0) and high perceived social mobility (low condition = 0, high condition = 1, control condition = 0). Social trust was added as the mediator and sense of gain was added as the dependent variable, with gender, age, monthly family income, and subjective social status included as covariates (see Table 7).

**Figure 5:**
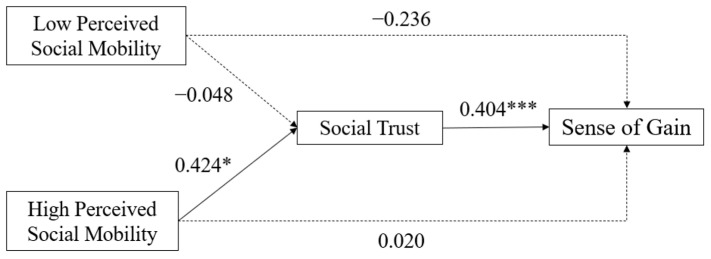
The mediation pathways of social trust (*n* = 188). Notes: All path coefficients are standardized; Solid arrows indicate significant paths, and dashed arrows indicate non-significant paths; * *p* < 0.05, *** *p* < 0.001.

The results of the overall mediation analysis indicate that the test for the total effect yields *F*_(2, 181)_ = 3.517, *p* = 0.032 < 0.05, suggesting that the two relative total effects are not all zero. The test for the overall direct effect yields *F*_(2, 180)_ = 1.544, *p* = 0.216, indicating that the two relative direct effects are not significant. Figure 5 illustrates the standardized coefficients of the model. There was neither a significant direct path from high perceived social mobility to sense of gain (β = 0.192, *p* = 0.364) nor a significant direct path from low perceived social mobility to sense of gain (β = −0.255, *p* = 0.209). However, there was a significant indirect path from high perceived social mobility to sense of gain through social trust (β = 0.172, 95% CI = [0.015, 0.349]). A non-significant indirect path was found from low perceived social mobility to sense of gain through social trust (β = − 0.020, 95% CI = [−0.192, 0.136]). Consistent with Study 1, this pattern again supports the hypothesized differential mechanism, whereby high perceived social mobility operates through social trust to enhance sense of gain, whereas this pathway is absent under low perceived social mobility. Monte Carlo simulation results showed that the indirect effect was significant, with estimated statistical power of 0.99, indicating a robust mediation effect.

#### 4.4.3. Analysis of Variance Test

**Figure 6:**
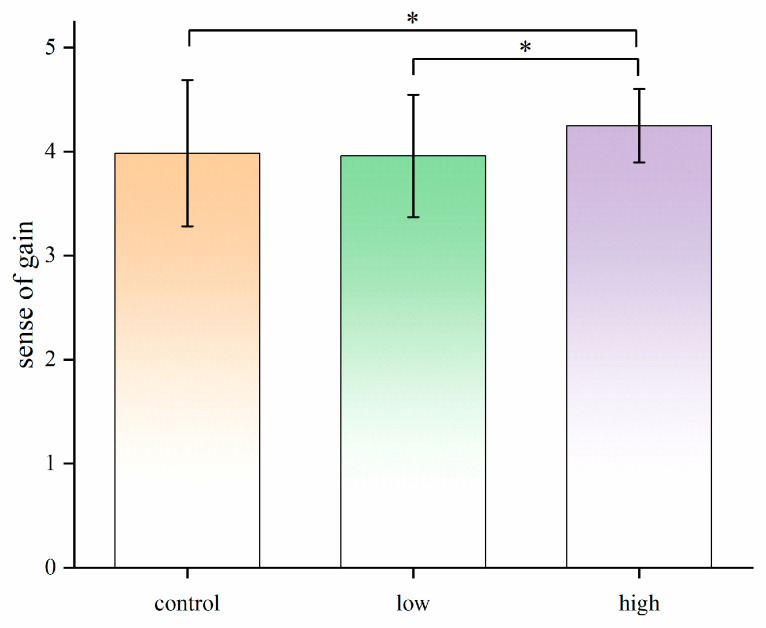
Differences in the sense of gain across perceived social mobility (*n* = 188). Notes: * *p* < 0.05. Error bars represent standard deviation (SD). Orange bars represent the control group, green bars represent the low perceived social mobility group, and purple bars represent the high perceived social mobility group.

A one-way analysis of variance was conducted to examine whether perceived social mobility would enhance social trust and sense of gain. The results indicated significant differences in sense of gain among the three groups, *F*_(2, 185)_ = 5.018, *p* < 0.01, η^2^ = 0.051. Participants in the high perceived social mobility group reported a significantly higher sense of gain (*M* = 4.25, *SD* = 0.354) compared to the control group (*M* = 3.99, *SD* = 0.704, *md* = 0.266, *p* < 0.05, 95% CI = [0.018, 0.512], Cohen’s *d* = 0.5), and significantly higher than the low perceived social mobility group (*M* = 3.96, *SD* = 0.590, *md* = 0.291, *p* < 0.05, 95% CI = [0.046, 0.536], Cohen’s *d* = 0.6). There was no significant difference in sense of gain between the control group and the low perceived social mobility group (*md* = 0.03, *p* > 0.05, 95% CI = [−0.219, 0.270]) (see Figure 6).

**Figure 7:**
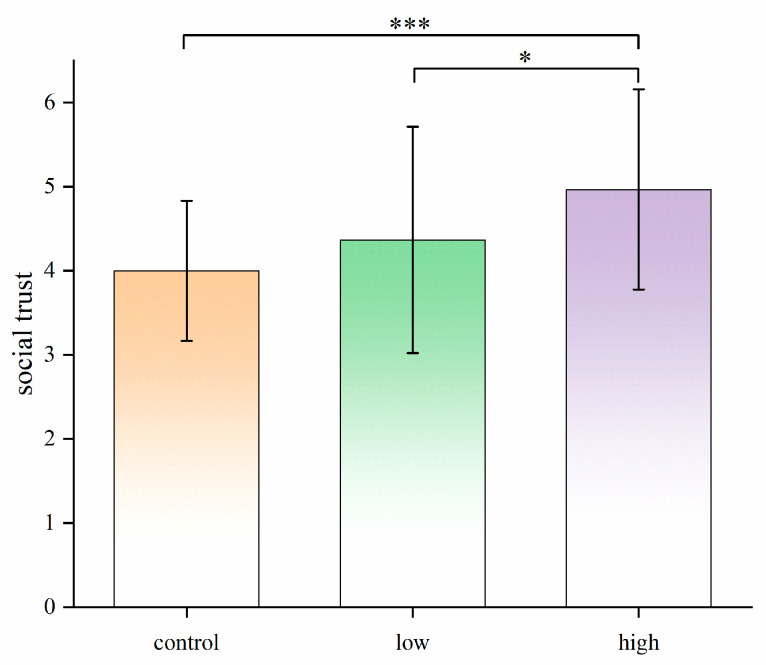
Differences in the social trust across perceived social mobility (*n* = 188). Notes: * *p* < 0.05, *** *p* < 0.001. Error bars represent standard deviation (SD). Orange bars represent the control group, green bars represent the low perceived social mobility group, and purple bars represent the high perceived social mobility group.

There were significant differences in social trust among the three groups, *F*_(2, 185)_ = 11.279, *p* < 0.001, η^2^ = 0.109. Participants in the high perceived social mobility group reported significantly higher social trust (*M* = 4.97, *SD* = 1.190) compared to the control group (*M* = 4.00, *SD* = 0.833, *md* = 0.97, *p* < 0.001, 95% CI = [0.471, 1.465], Cohen’s *d* = 1.3) and significantly higher than the low perceived social mobility group (*M* = 4.37, *SD* = 1.345, *md* = 0.60, *p* < 0.05, 95% CI = [0.107, 1.094], Cohen’s *d* = 1.2). There was no significant difference in social trust between the control group and the low perceived social mobility group (*md* = −0.37, *p* > 0.05, 95% CI = [−0.860, 0.126]) (see Figure 7).

#### 4.4.4. The Moderating Effect of Subjective Social Status

**Figure 8:**
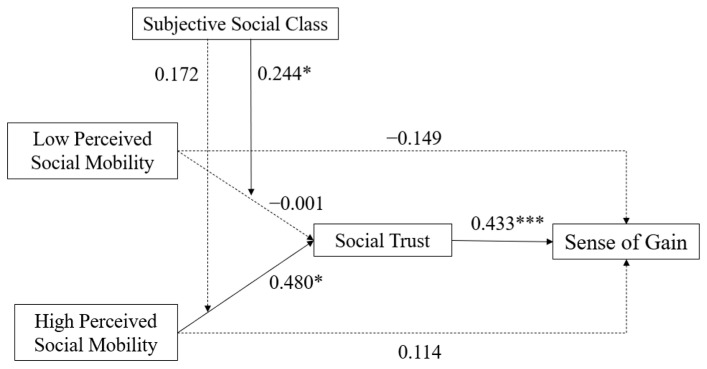
Moderated mediation path diagram (*n* = 188). Notes: All path coefficients are standardized; Solid arrows indicate significant paths, and dashed arrows indicate non-significant paths; * *p* < 0.05, *** *p* < 0.001.

Referring to the method for moderated mediation analysis with multiple categorical independent variables (Fang et al., 2023), the analysis was conducted using Model 7 of the PROCESS macro (version 3.3) with 5000 bootstrap samples. The coding method of the independent variables is consistent with the coding method used in the mediation analysis. Subjective social status served as the moderating variable, while social trust was added as the mediator and sense of gain was added as the dependent variable (see Table 8 and Figure 8).

**Figure 9:**
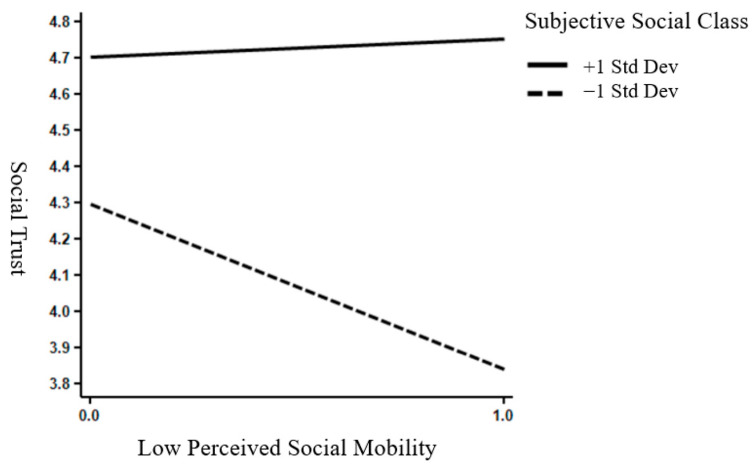
The moderating effect of subjective social status on low perceived social mobility and social trust.

When the subjective social status is low (Mean − 1SD = 3.54), the relative mediating effect of social trust regarding low perceived social mobility compared to the control group is −0.350, *SE* = 0.247, 95% CI = [−0.837, 0.137], indicating that the relative mediating effect is not statistically significant. When the subjective social status is moderate (Mean = 4.97), the relative mediating effect of social trust for low perceived social mobility compared to the control group is −0.001, *SE* = 0.197, 95% CI = [−0.389, 0.388], which is also not significant. When the subjective social status is high (Mean + 1SD = 6.40), the relative mediating effect of social trust for low perceived social mobility compared to the control group is 0.349, *SE* = 0.268, 95% CI = [−0.180, 0.877], again showing no significant effect. The difference in the mediating effect of social trust across levels of subjective social status is statistically significant (index of moderated mediation = 0.106, *SE* = 0.057, 95% CI = [0.008, 0.231]). Thus, it can be concluded that the relative mediating effect of social trust regarding low perceived social mobility is moderated by subjective social status (see Figure 9). However, the relative mediating effect of social trust for high perceived social mobility is not moderated by subjective social status (index of moderated mediation = 0.074, *SE* = 0.063, 95% CI = [−0.051, 0.203]). These findings indicate that subjective social status moderates the strength of the indirect effect, but the non-significant conditional effects at all levels of status preclude a firm conclusion that low status amplifies the negative pathway. The pattern points to a possible gradient but remains suggestive rather than definitive.

## 5. Discussion

This study shifts the focus of perceived social mobility in China from a “degree” perspective to a “category” perspective, revealing differential patterns and boundary conditions associated with high and low perceived mobility. The finding that perceived social mobility positively predicts sense of gain is consistent with previous research (Tan & Lv, 2023; Y. Wang et al., 2020). Extending beyond these studies, the present research shows that social trust mediates this relationship, with high perceived social mobility enhancing social trust and, in turn, sense of gain. Subjective social status also moderates the relationship between low perceived social mobility and social trust, with individuals of low subjective social status showing a stronger negative association between low perceived social mobility and social trust. This result suggests that the sense of gain dilemma experienced by individuals with low perceived social mobility cannot be resolved by simply replicating conventional approaches aimed at “strengthening mobility beliefs”. Instead, intervention designs need to shift toward a multi-pronged strategy that reconstructs the class reference framework and enhances resource visibility.

Perceived social mobility is indirectly associated with the sense of gain through social trust. This finding reflects the hierarchical nature of social mentality and underscores the role of social trust as an intermediary between foundational and higher-level processes (M. Chen, 2024). Individuals who perceive high social mobility tend to view society as open and fair and therefore extend trust to others. In high-trust environments, people are more likely to perceive social justice and warmth, which fosters positive emotions and a greater sense of gain (Helliwell et al., 2014). This pathway illustrates how a specific social axiom—the belief that effort is rewarded within an open social structure—can translate into tangible psychological benefits by shaping the relational context in which individuals pursue their goals.

The core finding of this study is that the indirect path from perceived social mobility to sense of gain via social trust was observed only among individuals reporting high mobility, and not among those reporting low mobility. While this difference is consistent with a resource conservation perspective, in which perceived constraints may prompt withdrawal from trust-based exchange (Hobfoll et al., 2018), the non-significant indirect effect in the low-mobility group allows for several interpretations. For instance, low perceived mobility might redirect trust toward more particularistic ties rather than generalized others, or it may activate direct affective responses that reduce sense of gain without necessarily operating through social trust. Future research should examine whether low perceived mobility operates through distinct mechanisms of its own.

The findings also suggest that subjective social status moderates the association between low perceived social mobility and social trust. The negative association between low perceived social mobility and social trust appears to be more pronounced among individuals with low subjective social status. This finding highlights an important feature of belief systems: beliefs do not operate in isolation. Rather, the psychological consequences of mobility-related beliefs may depend on how individuals position themselves within the social structure that those beliefs describe. When a negative axiom about mobility is combined with a low sense of personal standing, the two pieces of information converge on the same conclusion—that valued goals are unlikely to be attained—and social trust is correspondingly diminished. By contrast, subjective social status did not appear to moderate the association between high perceived social mobility and sense of gain, possibly because high mobility perceptions already provide a broadly affirmative outlook on future opportunities, leaving less room for subjective status to play a role. Prior research indicates that high perceived social mobility can lessen the psychological gap for those in lower social classes, thereby enhancing their sense of gain (Rao et al., 2022). In contrast, for individuals with low perceived social mobility, the resources and cognitive styles associated with different levels of subjective social statuses significantly shape perceptions of social trust (Kraus et al., 2009; Stephens et al., 2014).

Caution is especially warranted when interpreting the pattern among the dual-vulnerable group characterized by both low perceived social mobility and low subjective social status. Individuals in this group may be more likely to attribute stalled mobility to stable and uncontrollable external factors, fostering the belief that effort is ineffective. They may view limited resources as persistently constrained, reduce investments in social trust to preserve remaining resources, and thereby erode sense of gain as psychological resources diminish. Accordingly, interventions must go beyond universal solutions and address both cognitive and resource-related aspects. At the cognitive level, approaches such as psychological empowerment and attribution training can help weaken entrenched beliefs in “hopeless mobility” and support more positive and controllable self-positioning. At the resource level, the government and social organizations should collaborate to build accessible development resources and establish robust social support networks. For groups that already hold a high perception of social mobility, intervention should focus on further consolidating and amplifying its positive effects. By optimizing institutional environments and broadening upward pathways, individuals can be encouraged to transform positive mobility beliefs into sustained investment and development behavior, making them active carriers of social vitality.

Research indicates that from 2019 to 2022, the subjective social status of the Chinese public remained low, with no significant improvement (J. Wang & Zhang, 2023). This paradox suggests that a single income-based class framework cannot fully capture the complexity of subjective status perception. As social structure shifts from a pyramid to an olive shape, class evaluation benchmarks rise, while the lowest tier shrinks (Zhu & Han, 2024). Despite improvements in absolute terms, the expansion of the middle class has raised expectations for “middle status” identification, causing many individuals to feel relatively downwardly mobile. In addition, people often compare their weaknesses with others’ strengths, which lowers self-assessment. For example, those in stable jobs may undervalue their social prestige relative to business owners, who in turn may downplay their economic autonomy by emphasizing professional risks. To align subjective social status with objective well-being, it is necessary to rationalize reference standards and make implicit gains explicit.

This study helps fill a gap in the literature by examining perceived social mobility, social trust, and sense of gain within an integrated framework and by comparing high and low perceived mobility conditions rather than treating mobility perception as a purely linear variable. It also extends prior work by showing that subjective social status may condition the association between low perceived social mobility and social trust.

Several limitations should be acknowledged. First, Study 1 relied on a cross-sectional questionnaire survey, so common-method bias cannot be ruled out entirely. Second, the data were collected from online samples, which may limit sample representativeness and the generalizability of the findings. Third, the study focused exclusively on social trust as a mediator and did not examine other mechanisms that may operate when perceived social mobility is low. Fourth, although the index of moderated mediation was significant, the conditional indirect effects at specific levels of subjective social status did not consistently reach statistical significance. Thus, while the findings provide suggestive evidence for a moderating role of subjective social status, further replication is needed before stronger conclusions can be drawn. Finally, because the study was conducted in a Chinese cultural context, the cross-cultural generalizability of the findings may be limited.

These limitations also indicate several directions for future research. Future studies could adopt longitudinal, field-based, or diary-based survey designs to capture how naturally formed social mobility perceptions evolve over time and influence sense of gain in more ecologically valid settings. Moreover, future work should examine additional mediators beyond social trust, such as psychological resource depletion, perceived control, or emotional exhaustion, to provide a more comprehensive account of the underlying mechanism. With respect to moderation, future research should test whether the conditional indirect effect is robust across levels of subjective social status, and to clarify under what conditions the proposed boundary effect can be observed. Moreover, given that the national-level framing in Study 2 might also affect institutional trust or national optimism, future research should employ more neutral manipulations and include these variables as covariates. Finally, to address potential confounding variables in the experimental manipulation, future research should control for relevant factors such as mood and national optimism.

## 6. Conclusions

(1) Social trust mediates the relationship between perceived social mobility and sense of gain.
(2) When categorizing perceived social mobility as low or high, the indirect association through social trust differs across the two conditions. This indirect effect is significant under high perceived social mobility but not significant under low perceived social mobility.
(3) Subjective social status moderates the association between low perceived social mobility and social trust, with a tendency toward a more negative association at lower subjective social status levels.

## Figures and Tables

**Table 1:** Mean, standard deviation, and the correlation matrix of variables in study 1 (*n* = 372).

Variable	*M*	*SD*	1	2	3
1. Perceived Social Mobility	4.383	0.973	1		
2. Social Trust	4.505	1.222	0.530 ***	1	
3. Sense of Gain	5.412	0.805	0.479 ***	0.517 ***	1

Notes: *** *p* < 0.001. The data in the table were obtained through SPSS version 25.0 analysis based on the data collected by the authors.

**Table 2:** Analysis of the mediating effect of social trust in study 1 (*n* = 372).

Equation	Variables	*B*	*SE*	β	*t*	*p*	95% CI	*R*	*R* ^2^	*F*
**Equation 1 (Sense of Gain)**	Perceived social mobility	0.295	0.039	0.356	7.556 ***	0.000	[0.218, 0.371]	0.573	0.328	35.683 ***
	Gender	−0.070	0.069	−0.044	−1.012	0.313	[−0.206, 0.066]			
	Age	0.227	0.074	0.141	3.084 **	0.002	[0.082, 0.372]			
	Monthly family income	0.043	0.009	0.226	4.997 ***	0.000	[0.026, 0.060]			
	Subjective social status	0.057	0.025	0.108	2.308 *	0.022	[0.008, 0.105]			
**Equation 2 (Social Trust)**	Perceived social mobility	0.528	0.059	0.420	9.012 ***	0.000	[0.413, 0.643]	0.586	0.343	38.199 ***
	Gender	0.015	0.104	0.006	0.145	0.885	[−0.190, 0.220]			
	Age	0.254	0.110	0.104	2.295 *	0.022	[0.036, 0.471]			
	Monthly family income	0.016	0.013	0.054	1.212	0.226	[−0.010, 0.041]			
	Subjective social status	0.166	0.037	0.207	4.486 ***	0.000	[0.093, 0.239]			
**Equation 3 (Sense of Gain)**	Perceived social mobility	0.189	0.041	0.228	4.582 ***	0.000	[0.108, 0.269]	0.624	0.389	38.732 ***
	Social trust	0.201	0.033	0.305	6.051 ***	0.000	[0.136, 0.266]			
	Gender	−0.073	0.066	−0.045	−1.105	0.270	[−0.203, 0.057]			
	Age	0.176	0.071	0.110	2.487 **	0.013	[0.037, 0.315]			
	Monthly family income	0.040	0.008	0.209	4.841 ***	0.000	[0.024, 0.056]			
	Subjective social status	0.024	0.024	0.044	0.972	0.332	[−0.024, 0.071]			

Notes: * *p* < 0.05, ** *p* < 0.01, *** *p* < 0.001. The data in the table were obtained through SPSS version 25.0 analysis based on the data collected by the authors.

**Table 3:** The mediation analysis in study 1 (*n* = 372).

Equation	Variables	*B*	*SE*	β	*t*	*p*	95% CI	*R*	*R* ^2^	*F*
**Equation 1 (Social Trust)**	Low perceived social mobility	−0.207	0.134	−0.169	−1.550	0.122	[−0.470, 0.056]	0.575	0.330	29.986 ***
	High perceived social mobility	1.019	0.138	0.833	7.400 ***	0.000	[0.748, 1.289]			
	Gender	0.007	0.105	0.003	0.062	0.951	[−0.200, 0.214]			
	Age	0.158	0.117	0.065	1.352	0.177	[−0.072, 0.387]			
	Monthly family income	0.015	0.013	0.052	1.135	0.257	[−0.011, 0.041]			
	Subjective social status	0.183	0.038	0.228	4.825 ***	0.000	[0.109, 0.258]			
**Equation 2 (Sense of Gain)**	Low perceived social mobility	−0.039	0.084	−0.048	−0.459	0.647	[−0.204, 0.127]	0.626	0.392	33.574 ***
	High perceived social mobility	0.414	0.093	0.514	4.466 ***	0.000	[0.232, 0.596]			
	Social trust	0.202	0.033	0.307	6.148 ***	0.000	[0.137, 0.267]			
	Gender	−0.077	0.066	−0.048	−1.170	0.243	[−0.207, 0.053]			
	Age	0.127	0.073	0.079	1.730	0.085	[−0.017, 0.271]			
	Monthly family income	0.039	0.008	0.204	4.708 ***	0.000	[0.023, 0.055]			
	Subjective social status	0.030	0.025	0.057	1.226	0.221	[−0.018, 0.078]			

Notes: *** *p* < 0.001. The data in the table were obtained through SPSS version 25.0 analysis based on the data collected by the authors.

**Table 4:** Mean, standard deviation, and the correlation matrix of variables in pre-experiment (*n* = 44).

Variable	*M*	*SD*	1	2	3	4	5
1. Perceived Social Mobility	0.50	0.506	1				
2. Belief In a Just World	5.458	1.045	0.161	1			
3. Relative Deprivation	2.949	0.110	−0.088	−0.553 ***	1		
4. Risk Perception	4.383	1.481	−0.195	−0.331 *	0.609 ***	1	
5. Social Expectation	5.125	1.015	0.147	0.656 ***	−0.406 **	−0.191	1

Notes: * *p* < 0.05, ** *p* < 0.01, *** *p* < 0.001. The data in the table were obtained through SPSS version 25.0 analysis based on the data collected by the authors.

**Table 5:** Mean, standard deviation, and the correlation matrix of variables in study 2 (*n* = 188).

Variables	*M*	*SD*	1	2	3
**1. Perceived Social Mobility**	1	0.814	1		
**2. Social Trust**	4.44	1.207	0.326 ***	1	
**3. Sense of Gain**	4.06	0.581	0.186 *	0.435 ***	1

Notes: Control group = 0, low perceived social mobility group = 1, high perceived social mobility group = 2. * *p* < 0.05, *** *p* < 0.001. The data in the table were obtained through SPSS version 25.0 analysis based on the data collected by the authors.

**Table 6:** Analysis of the mediating effect of social trust in study 2 (*n* = 126).

Equation	Variables	*B*	*SE*	β	*t*	*p*	95% CI	*R*	*R* ^2^	*F*
**Equation 1 (Sense of Gain)**	Perceived social mobility	0.269	0.086	0.530	3.138 **	0.002	[0.099, 0.439]	0.394	0.155	4.402 ***
	Gender	0.103	0.097	0.092	1.064	0.289	[−0.089, 0.295]			
	Age	0.072	0.070	0.089	1.018	0.311	[−0.068, 0.211]			
	Monthly family income	0.004	0.018	0.019	0.221	0.825	[−0.031, 0.039]			
	Subjective social status	0.100	0.038	0.228	2.651 **	0.009	[0.025, 0.174]			
**Equation 2 (Social Trust)**	Perceived social mobility	0.549	0.217	0.422	2.527 *	0.013	[0.119, 0.979]	0.420	0.176	5.131 ***
	Gender	0.104	0.246	0.036	0.424	0.672	[−0.382, 0.590]			
	Age	0.510	0.178	0.246	2.862 **	0.005	[0.157, 0.863]			
	Monthly family income	0.019	0.045	0.036	0.425	0.672	[−0.069, 0.107]			
	Subjective social status	0.237	0.095	0.211	2.483 *	0.014	[0.048, 0.425]			
**Equation 3 (Sense of Gain)**	Perceived social mobility	0.158	0.076	0.311	2.080 *	0.040	[0.008, 0.308]	0.615	0.378	12.061 ***
	Social trust	0.203	0.031	0.520	6.535 ***	0.000	[0.142, 0.265]			
	Gender	0.082	0.084	0.073	0.982	0.328	[−0.084, 0.248]			
	Age	−0.032	0.063	−0.040	−0.509	0.612	[−0.156, 0.092]			
	Monthly family income	0.0001	0.015	0.0002	0.003	0.997	[−0.030, 0.030]			
	Subjective social status	0.052	0.033	0.118	1.556	0.122	[−0.014, 0.118]			

Notes: * *p* < 0.05, ** *p* < 0.01, *** *p* < 0.001. The data in the table were obtained through SPSS version 25.0 analysis based on the data collected by the authors.

**Table 7:** The mediation analysis in study 2 (*n* = 188).

Equation	Variables	*B*	*SE*	β	*t*	*p*	95% CI	*R*	*R* ^2^	*F*
**Equation 1 (Social Trust)**	Low perceived social mobility	−0.058	0.238	−0.048	−0.246	0.806	[−0.527, 0.411]	0.423	0.179	6.566 ***
	High perceived social mobility	0.512	0.247	0.424	2.071 *	0.040	[0.024, 1.000]			
	Gender	−0.062	0.178	−0.025	−0.348	0.729	[−0.413, 0.290]			
	Age	0.515	0.160	0.263	3.220 **	0.002	[0.199, 0.830]			
	Monthly family income	0.002	0.024	0.005	0.067	0.947	[−0.046, 0.050]			
	Subjective social status	0.110	0.060	0.131	1.827	0.069	[−0.009, 0.230]			
**Equation 2 (Sense of Gain)**	Low perceived social mobility	−0.137	0.109	−0.236	−1.261	0.209	[−0.351, 0.077]	0.514	0.264	9.240 ***
	High perceived social mobility	0.012	0.114	0.020	0.103	0.910	[−0.214, 0.237]			
	Social trust	0.195	0.034	0.404	5.733 ***	0.000	[0.128, 0.262]			
	Gender	0.076	0.081	0.064	0.930	0.354	[−0.085, 0.236]			
	Age	−0.040	0.075	−0.043	−0.536	0.593	[−0.188, 0.108]			
	Monthly family income	−0.028	0.011	−0.164	−2.507 *	0.013	[−0.050, −0.006]			
	Subjective social status	0.083	0.028	0.204	2.982 **	0.003	[0.028, 0.138]			

Notes: * *p* < 0.05, ** *p* < 0.01, *** *p* < 0.001. The data in the table were obtained through SPSS version 25.0 analysis based on the data collected by the authors.

**Table 8:** The moderated mediation analysis in study 2 (*n* = 188).

Equation	Variables	*B*	*SE*	β	*t*	*p*	95% CI	*R*	*R* ^2^	*F*
**Equation 1 (Social Trust)**	Low perceived social mobility	−0.001	0.197	−0.001	−0.003	0.998	[−0.389, 0.388]	0.448	0.201	5.621 ***
	High perceived social mobility	0.579	0.207	0.480	2.317 *	0.022	[0.071, 0.889]			
	Subjective social status	−0.012	0.069	−0.010	−0.150	0.881	[−0.145, 0.125]			
	INTI 1	0.295	0.116	0.244	2.106 *	0.037	[0.015, 0.473]			
	INTI 2	0.207	0.128	0.172	1.337	0.183	[−0.082, 0.425]			
	Gender	−0.051	0.147	−0.043	−0.290	0.772	[−0.333, 0.247]			
	Age	0.474	0.132	0.393	2.969 **	0.003	[0.132, 0.654]			
	Monthly family income	0.003	0.020	0.002	0.122	0.903	[−0.037, 0.042]			
**Equation 2 (Sense of Gain)**	Low perceived social mobility	−0.087	0.189	−0.149	−0.790	0.431	[−0.521, 0.223]	0.478	0.228	8.910 ***
	High perceived social mobility	0.066	0.199	0.114	0.572	0.568	[−0.278, 0.505]			
	Social trust	0.208	0.071	0.433	6.059 ***	0.000	[0.292, 0.574]			
	Gender	0.091	0.143	0.156	1.092	0.276	[−0.126, 0.438]			
	Age	−0.036	0.132	−0.062	−0.467	0.641	[−0.322, 0.199]			
	Monthly family income	−0.023	0.019	−0.039	−2.025 *	0.044	[−0.077, −0.001]			

Notes: * *p* < 0.05, ** *p* < 0.01, *** *p* < 0.001. INTI 1 represents the interaction between low perceived social mobility and subjective social status. INTI 2 represents the interaction between high perceived social mobility and subjective social status. *B* values are unstandardized coefficients. All other values (e.g., β, *SE*, 95%CI) are standardized. The data in the table were obtained through SPSS version 25.0 analysis based on the data collected by the authors.

## Data Availability

The original data presented in the study are openly available in Figshare at https://doi.org/10.6084/m9.figshare.30971692.

## Supplementary Materials

The following supporting information can be downloaded at: https://www.mdpi.com/article/10.3390/bs16071141/s1, the supplementary materials include the full item wording for all measurement scales used in this study.

## Appendix A

**Table A1:** Characteristics of the participants in study 1 (*n* = 372).

Variable	Category	Number	Percent	Range
Gender	male	174	46.8	
	female	198	53.2	
Age	18–25 years	277	74.5	1–3
	26–35 years	86	23.1	
	36–45 years	9	2.4	
Educational level	Primary school or below	0	0	1–6
	Middle school	1	0.3	
	Senior high school	4	1.1	
	Associate degree	12	3.2	
	Bachelor’s degree	312	83.9	
	Postgraduate degree or higher	43	11.6	
Household registration type	Local urban	127	34.1	1–4
	Local rural	90	24.2	
	Non-local urban	43	11.6	
	Non-local rural	112	30.1	
Monthly family income	1000 yuan or below	3	0.8	1–19
	1001–2000 yuan	7	1.9	
	2001–3000 yuan	9	2.4	
	3001–4000 yuan	17	4.6	
	4001–5000 yuan	17	4.6	
	5001–6000 yuan	20	5.4	
	6001–7000 yuan	21	5.6	
	7001–8000 yuan	26	7.0	
	8001–9000 yuan	15	4.0	
	9001–10,000 yuan	27	7.3	
	10,001–15,000 yuan	57	15.3	
	15,001–20,000 yuan	46	12.4	
	20,001–25,000 yuan	40	10.8	
	25,001–30,000 yuan	12	3.2	
	30,001–40,000 yuan	12	3.2	
	40,001–50,000 yuan	4	1.1	
	50,001–70,000 yuan	11	3.0	
	70,001–100,000 yuan	6	1.6	
	100,000 yuan and above	22	5.9	

**Table A2:** Characteristics of the participants in study 2 (*n* = 188).

Variable	Category	Number	Percent	Range
Gender	male	72	38.3	
	female	116	61.7	
Age	18–25 years	108	57.4	1–3
	26–35 years	68	36.2	
	36–45 years	12	6.4	
Educational level	Primary school or below	0	0	1–6
	Middle school	0	0	
	Senior high school	2	1.1	
	Associate degree	10	5.3	
	Bachelor’s degree	161	85.6	
	Postgraduate degree or higher	15	8.0	
Household registration type	Local urban	72	38.3	1–4
	Local rural	37	19.7	
	Non-local urban	26	13.8	
	Non-local rural	53	28.2	
Monthly family income	1000 yuan or below	2	1.1	1–19
	1001–2000 yuan	1	.5	
	2001–3000 yuan	8	4.3	
	3001–4000 yuan	8	4.3	
	4001–5000 yuan	11	5.9	
	5001–6000 yuan	5	2.7	
	6001–7000 yuan	3	1.6	
	7001–8000 yuan	11	5.9	
	8001–9000 yuan	8	4.3	
	9001–10,000 yuan	20	10.6	
	10,001–15,000 yuan	80	42.6	
	15,001–20,000 yuan	13	6.9	
	20,001–25,000 yuan	4	2.1	
	25,001–30,000 yuan	3	1.6	
	30,001–40,000 yuan	1	.5	
	40,001–50,000 yuan	2	1.1	
	50,001–70,000 yuan	2	1.1	
	70,001–100,000 yuan	2	1.1	
	100,000 yuan and above	4	2.1	

## References

[B1-behavsci-16-01141] Adler N. E., Epel E. S., Castellazzo G., Ickovics J. R. (2000). Relationship of subjective and objective social status with psychological and physiological functioning: Preliminary data in healthy, white women. Health Psychology.

[B2-behavsci-16-01141] Barbareschi G., Sanderman R., Kempen G. I., Ranchor A. V. (2008). The mediating role of perceived control on the relationship between socioeconomic status and functional changes in older patients with coronary heart disease. The Journals of Gerontology Series B: Psychological Sciences and Social Sciences.

[B3-behavsci-16-01141] Bond M. H., Leung K., Au A., Tong K. K., De Carrasquel S. R., Murakami F., Lewis J. R. (2004). Culture-level dimensions of social axioms and their correlates across 41 cultures. Journal of Cross-Cultural Psychology.

[B4-behavsci-16-01141] Brandt M. J., Wetherell G., Henry P. J. (2015). Changes in income predict change in social trust: A longitudinal analysis. Political Psychology.

[B5-behavsci-16-01141] Chen H., Wang P., Hao S. (2025). AI in the spotlight: The impact of artificial intelligence disclosure on user engagement in short-form videos. Computers in Human Behavior.

[B6-behavsci-16-01141] Chen M. (2024). Fluidity mindset and societal mentality.

[B7-behavsci-16-01141] Cramer E. M., Song H., Drent A. M. (2016). Social comparison on Facebook: Motivation, affective consequences, self-esteem, and Facebook fatigue. Computers in Human Behavior.

[B8-behavsci-16-01141] Dalbert C. (1999). The world is more just for me than generally: About the personal belief in a just world scale’s validity. Social Justice Research.

[B9-behavsci-16-01141] Davidai S., Wienk M. N. (2021). The psychology of lay beliefs about economic mobility. Social and Personality Psychology Compass.

[B10-behavsci-16-01141] Day M. V., Fiske S. T. (2017). Movin’on up? How perceptions of social mobility affect our willingness to defend the system. Social Psychological and Personality Science.

[B11-behavsci-16-01141] Delhey J., Newton K., Welzel C. (2011). How general is trust in “most people”? Solving the radius of trust problem. American Sociological Review.

[B12-behavsci-16-01141] Demakakos P., Nazroo J., Breeze E., Marmot M. (2008). Socioeconomic status and health: The role of subjective social status. Social Science & Medicine.

[B13-behavsci-16-01141] Dohrenwend B. S., Dohrenwend B. P. (1981). Socioenvironmental factors, stress, and psychopathology. American Journal of Community Psychology.

[B14-behavsci-16-01141] Dong H., Tan X., Dou X., Wang J. (2019). The structure of Chinese people’s sense of gain. Psychological Exploration.

[B15-behavsci-16-01141] Dong Y., Wang X. (2025). What if hard work cannot pay off? Perceived low social mobility increases passive procrastination among students. Social Psychological and Personality Science.

[B16-behavsci-16-01141] Fang J., Wen Z., He Z. (2023). Analysis of moderated mediation models with a categorical moderator. Chinese Journal of Applied Psychology.

[B17-behavsci-16-01141] Fang J., Wen Z., Zhang M. (2017). Analysis of mediation effects with categorical variables. Journal of Psychological Science.

[B18-behavsci-16-01141] Feng L., Zhong H. (2021). Interrelationships and methods for improving university students’ sense of gain, sense of security, and happiness. Frontiers in Psychology.

[B19-behavsci-16-01141] Gallo L. C., Matthews K. A. (2003). Understanding the association between socioeconomic status and physical health: Do negative emotions play a role?. Psychological Bulletin.

[B20-behavsci-16-01141] Gugushvili A. (2022). Information about inequality of opportunity increases downward mobility perceptions: A population-wide randomized survey experiment. Frontiers in Psychology.

[B21-behavsci-16-01141] Hamamura T. (2012). Social class predicts generalized trust but only in wealthy societies. Journal of Cross-Cultural Psychology.

[B22-behavsci-16-01141] Hayes A. F. (2013). Introduction to mediation, moderation, and conditional process analysis: A regression-based approach.

[B23-behavsci-16-01141] Helliwell J. F., Huang H., Wang S. (2014). Social capital and well-being in times of crisis. Journal of Happiness Studies.

[B24-behavsci-16-01141] Hobfoll S. E. (1989). Conservation of resources: A new attempt at conceptualizing stress. American Psychologist.

[B25-behavsci-16-01141] Hobfoll S. E., Halbesleben J., Neveu J. P., Westman M. (2018). Conservation of resources in the organizational context: The reality of resources and their consequences. Annual Review of Organizational Psychology and Organizational Behavior.

[B26-behavsci-16-01141] Huang S., Hou J., Sun L., Dou D., Liu X., Zhang H. (2017). The effects of objective and subjective socioeconomic status on subjective well-being among rural-to-urban migrants in China: The moderating role of subjective social mobility. Frontiers in Psychology.

[B27-behavsci-16-01141] Jackson B., Richman L. S., LaBelle O., Lempereur M. S., Twenge J. M. (2015). Experimental evidence that low social status is most toxic to well-being when internalized. Self and Identity.

[B28-behavsci-16-01141] Ji C., Hu Y. (2022). Economic development and longitudinal sense of gain: An analysis based on global panel data. Journal of Public Administration.

[B29-behavsci-16-01141] Kraus M. W., Keltner D. (2013). Social class rank, essentialism, and punitive judgment. Journal of Personality and Social Psychology.

[B30-behavsci-16-01141] Kraus M. W., Piff P. K., Keltner D. (2009). Social class, sense of control, and social explanation. Journal of Personality and Social Psychology.

[B31-behavsci-16-01141] Kraus M. W., Piff P. K., Mendoza-Denton R., Rheinschmidt M. L., Keltner D. (2012). Social class, solipsism, and contextualism: How the rich are different from the poor. Psychological Review.

[B32-behavsci-16-01141] Leung K., Bond M. H. (2004). Social axioms: A model for social beliefs in multicultural perspective. Advances in Experimental Social Psychology.

[B33-behavsci-16-01141] Li L., Shi L., Zhu B. (2018). A solidifying or mobile society? An analysis of China’s class structure over the past forty years. Sociological Studies.

[B34-behavsci-16-01141] Li W., Wu J., Kou Y. (2020). System justification enhances life satisfaction of high-and low-status people in China. Social Psychological and Personality Science.

[B35-behavsci-16-01141] Lin L., Hua L., Li J. (2022). Seeking pleasure or growth? The mediating role of happiness motives in the longitudinal relationship between social mobility beliefs and well-being in college students. Personality and Individual Differences.

[B36-behavsci-16-01141] Liu Z., Han G., Yan J., Liu Z., Osmani M. (2022). The relationship between social mentality and health in promoting well-being and sustainable city. International Journal of Environmental Research and Public Health.

[B37-behavsci-16-01141] Lu H., Tong P., Zhu R. (2020). Longitudinal evidence on social trust and happiness in China: Causal effects and mechanisms. Journal of Happiness Studies.

[B38-behavsci-16-01141] Luhmann N. (1979). Trust and power.

[B39-behavsci-16-01141] Ma A. (2012). Relative deprivation and social adaptation styles: Mediating and moderating effects. Acta Psychologica Sinica.

[B40-behavsci-16-01141] Madigan A., Daly M. (2023). Socioeconomic status and depressive symptoms and suicidality: The role of subjective social status. Journal of Affective Disorders.

[B41-behavsci-16-01141] Main G., Bradshaw J. (2012). A child material deprivation index. Child Indicators Research.

[B42-behavsci-16-01141] Navarro-Carrillo G., Valor-Segura I., Moya M. (2018). Do you trust strangers, close acquaintances, and members of your ingroup? Differences in trust based on social class in Spain. Social Indicators Research.

[B43-behavsci-16-01141] O’Leary D., Uysal A., Rehkopf D. H., Gross J. J. (2021). Subjective social status and physical health: The role of negative affect and reappraisal. Social Science & Medicine.

[B44-behavsci-16-01141] Putnam H. (1995). Philosophical papers: Mind, language and reality.

[B45-behavsci-16-01141] Rao T. T., Yang S. L., Yu F., Xu B. X., Wei J. (2022). Perception of class mobility moderates the relationship between social class and prosocial behaviour. Asian Journal of Social Psychology.

[B46-behavsci-16-01141] Ren Z., Yue G., Xiao W., Fan Q. (2022). The influence of subjective socioeconomic status on life satisfaction: The chain mediating role of social equity and social trust. International Journal of Environmental Research and Public Health.

[B47-behavsci-16-01141] Rheinschmidt M. L., Mendoza-Denton R. (2014). Social class and academic achievement in college: The interplay of rejection sensitivity and entity beliefs. Journal of Personality and Social Psychology.

[B48-behavsci-16-01141] Sagioglou C., Forstmann M., Greitemeyer T. (2019). Belief in social mobility mitigates hostility resulting from disadvantaged social standing. Personality and Social Psychology Bulletin.

[B49-behavsci-16-01141] Sakurai K., Kawakami N., Yamaoka K., Ishikawa H., Hashimoto H. (2010). The impact of subjective and objective social status on psychological distress among men and women in Japan. Social Science & Medicine.

[B50-behavsci-16-01141] Schilke O., Reimann M., Cook K. S. (2021). Trust in social relations. Annual Review of Sociology.

[B51-behavsci-16-01141] Schneider S. M. (2012). Income inequality and its consequences for life satisfaction: What role do social cognitions play?. Social Indicators Research.

[B52-behavsci-16-01141] Shao Y. (2019). Subjective sense of gain in the new era: Indicator development and analysis of influencing factors. Social Sciences in Xinjiang.

[B53-behavsci-16-01141] Stephens N. M., Markus H. R., Phillips L. T. (2014). Social class culture cycles: How three gateway contexts shape selves and fuel inequality. Annual Review of Psychology.

[B54-behavsci-16-01141] Su Z. Q., Zhang D. J., Wang X. Q. (2012). Revision of the Just World Belief Scale and its reliability and validity among college students. Chinese Journal of Behavioral Medicine and Brain Science.

[B55-behavsci-16-01141] Tan X. (2016). The effect of objective social status and subjective social status on social trust. Journal of Harbin Institute of Technology (Social Sciences Edition).

[B56-behavsci-16-01141] Tan X., Dong H., Zhang Y., Wang J. (2020). The concept, structure, and life satisfaction effects of the sense of gain. Sociological Studies.

[B57-behavsci-16-01141] Tan X., Lv M. (2023). Perceived social mobility and sense of gain among youth. Youth Studies.

[B58-behavsci-16-01141] Tang D., Wen Z. (2020). Testing for common method bias: Issues and recommendations. Journal of Psychological Science.

[B59-behavsci-16-01141] Wang J., Zhang Y. (2023). The social mentality foundation for common prosperity. Journal of the Party School of the Central Committee of the C.P.C..

[B60-behavsci-16-01141] Wang Y., Yang C., Hu X., Chen H. (2020). The mediating effect of community identity between socioeconomic status and sense of gain in Chinese adults. International Journal of Environmental Research and Public Health.

[B61-behavsci-16-01141] Xiang J., Ma Y. (2022). Spiritual common prosperity for the people: Its contemporary connotation, hierarchical structure, and pathways to attainment. Ideological & Theoretical Education.

[B62-behavsci-16-01141] Xiao L., Yao M., Liu H. (2024). Perceived social mobility and smartphone dependence in university students: The roles of hope and family socioeconomic status. Psychology Research and Behavior Management.

[B63-behavsci-16-01141] Xie J., Zhang B., Yao Z., Peng B., Chen H., Gao J. (2022). The relationship between social mobility belief and learning engagement in adolescents: The role of achievement goal orientation and psychological capital. Frontiers in Psychology.

[B64-behavsci-16-01141] Yang S., Guo Y., Hu X., Shu S., Li J. (2016). Do the lower class exhibit a higher level of system justification? An investigation from the social cognitive perspective. Acta Psychologica Sinica.

[B65-behavsci-16-01141] Yang Y., Ni Y. (2025). How does internet use affect the sense of gain in older adults? A moderated mediation model. Frontiers in Psychology.

[B66-behavsci-16-01141] Ye X., Li C. (2025). Do “flops” enhance authenticity? The impact of influencers’ proactive disclosures of failures on product recommendations. Behavioral Sciences.

[B67-behavsci-16-01141] Zhao S., Du H., King R. B., Lin D., Chi P. (2025). Growth mindset of socioeconomic status boosts academic-related outcomes. Social Psychology of Education.

[B68-behavsci-16-01141] Zhu G., Han L. (2024). Ten new trends in the evolution of China’s contemporary class structure. Administrative Tribune.

