# Supplementary material for: Differential Effect of Perceived Social Mobility on Sense of Gain
Source: Behav Sci (Basel). 2026 Jul 7;16(7):1141. doi: 10.3390/bs16071141 (PMC13406029; doi:10.3390/bs16071141)

Perceived social mobility

Please indicate your level of agreement with the following statements and select the corresponding option. (1 = Strongly Disagree; 2 = Disagree; 3 = Somewhat Disagree; 4 = Neutral; 5 = Somewhat Agree; 6 = Agree; 7 = Strongly Agree)

1. The social environment we are born into determines our entire life.
2. In today’s society, it is very difficult for a person to improve their social status throughout their life.
3. A person’s social status can easily decline.
4. In today’s society, the possibility of "starting from scratch" is still quite high.
5. Most social stratification systems are rigid and rarely change.
6. In today’s society, the wealthy can lose everything and become poor.
7. In today’s society, a person can improve their social class through hard work.

Sense of gain (study 1)

This questionnaire mentions some feelings of individuals. Please select the corresponding option based on your experience. (1 = Strongly Disagree; 2 = Disagree; 3 = Somewhat Disagree; 4 = Neutral; 5 = Somewhat Agree; 6 = Agree; 7 = Strongly Agree)

1. The social security system has alleviated many of my worries.
2. The inclusiveness and friendliness of society make my life more fulfilling.
3. A safe social environment allows me to live with peace of mind.
4. I have the means to travel.
5. I have a decent income.
6. I often get to reunite with my family.
7. My current life is very comfortable.
8. When I think about what I have now, I feel fulfilled.
9. I am content with what I currently have.
10. I have always been striving for a better life.
11. I constantly find ways to utilize my talents.
12. I actively seize opportunities to improve my life.
13. The collective well-being of society is more valuable than individual gain.
14. I am grateful to those who have helped me.
15. When a person achieves success, they should think about benefiting others.

Sense of gain (study 2)

In the past five years, what changes have you observed regarding the achievements of our country's reform and opening-up, as well as local development and construction? (1 = Experienced serious deterioration; 2 = Experienced some deterioration; 3 = Felt little change; 4 = Experienced slight improvement; 5 = Experienced significant improvement)

1. Anti-corruption and integrity promotion
2. Government service transparency
3. Rule of law construction
4. National economic development
5. Family income levels
6. Reforms in education equity

7. Social security reforms
8. Improvements in public transportation
9. Development of cultural activities
10. Protection and development of culture
11. Construction of cultural infrastructure
12. Environmental protection in communities or villages
13. Strengthening of ecological environment protection
14. Management of environmental pollution
15. Promotion and awareness of ecological civilization

Social trust

Please select the corresponding option based on your level of agreement with the following statements. (1 = Strongly Disagree; 2 = Disagree; 3 = Somewhat Disagree; 4 = Neutral; 5 = Somewhat Agree; 6 = Agree; 7 = Strongly Agree)

1. People are, in most cases: A. Helpful; B. Self-interested and indifferent to others.
2. Most people in society: A. Can be trusted; B. Should be approached with caution.
3. Most people in society will: A. Treat others as fairly as possible; B. Take advantage of others whenever possible.
4. Most people in society trust strangers.

Subjective social status

In our society, some people are at the upper levels, while others are at the lower levels. As illustrated in the diagram, the ladder viewed from top to bottom assigns a score of 10 to the highest level and a score of 1 to the lowest level.

For example, level 1 represents the very bottom of the social ladder, where people experience the worst living conditions—the lowest level of education, the least decent jobs, and the lowest income. Level 10 represents the very top of the social ladder, where people enjoy the most affluent living conditions—high levels of education, the most decent jobs, and the highest income.

Which level do you think you currently occupy? (1-10)

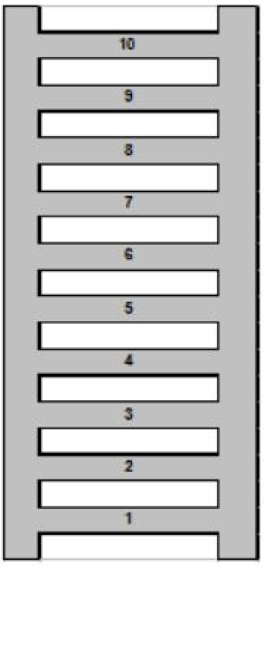

Supplement: Supplementary file 1 [file behavsci-16-01141-s001.zip › behavsci-4316725-supplementary.pdf]
